# Hospital Meetings, &c.

**Published:** 1896-12-19

**Authors:** 


### HOSPITAL MEETINGS, &C.

### METROPOLITAN HOSPITAL SUNDAY FUND.

The annual general meeting of constituents of the above fund was held at the Old Council Chamber, Guildhall, E.C., on Monday, 14th inst. In the unavoidable absence of the Lord Mayor, who was to have presided, Archdeacon Sinclair was voted to the chair.

The Bishop of Stepney proposed "That the report of the Council for the year 1896 is hereby received and approved." It was, he said, a great pleasure to propose the adoption of a report which was in all respects so satisfactory. With the exception of last year, which was in all ways an exceptional one, the total receipts had never, till 1896, reached £46,000, and the number of contributing congregations had never been so large; in fact, since 1873 this number had been steadily increasing, and it had now reached a total of 1,811. Further, the amounts received from congregations had never reached so large a total as this year. When they looked into this increase in the number of congregations one very satisfactory point was apparent. In the abstract people might say that of course the large and wealthy congregations all joined in in the first year, and the increase had been caused by small congregations coming in. It might also be supposed that the fund was started with great enthusiasm, and great efforts were made at the beginning. Further, it must be remembered that in the year 1873, property of many kinds was in a much more flourishing condition than at the present time. On these and other accounts one might expect that the average sum contributed by each congregation would show a considerable reduction; but whereas in the first year the average sum collected was £24 2s., it was now £22 7s. (Applause.) That the rate was so well maintained was very satisfactory in itself, and was a real assurance for the future. (Renewed applause.) He had frequently seen and heard the remark made that the Church of England's contributions were out of all proportion to those of other denominations. So far as this fact was used to the credit of the Church of England or the discredit of any other denominations, it was used most unjustly and unfairly, and he always protested against it. (Applause.) The enormous numbers of the members of the Church of England, the great wealth of many of them, and the fact that other denominations had not the endowments to maintain their ministers, would render it indeed disgraceful to the Church of England if there were not this apparent disproportion. (Applause.) He would enter into no comparison of that kind, but it was interesting to notice how many denominations were able to rise to a really important amount in the highest contributions made by any one congregation. No less than eight religious denominations gave the fund this year as the highest amount contributed by any one congregation more than £100, viz.: the Church of England, £1,508; Jews, £257; Unitarians, £131 17s.; Church of Scotland, £131; Congregationalists, £126; Presbyterians, £125; Baptists, £115; and the Greek Church, £104. Three denominations rose to between £50 and £100, viz.: the Dutch Protestants, £90; the German Lutherans, £68; and the Roman Catholics, £54. (Applause.) While mentioning these largest sums, it came into his mind that he had heard unfavourable criticism of the smallness of some of the amounts contributed by congregations. To him these smallest sums were the most pathetic features of the whole list of contributions. (Hear, hear.) He knew so well the poverty of the congregations of some of the mission churches of his own districts who were among these small contributors; he knew so well that there was not among them a person who ought to give more than a penny, and that every penny they could afford was wanted for the maintenance of their services and the decency of their mission room—(hear, hear)—that he was convinced that a sum of 15s. from a congregation of this kind was in fact a larger gift than £100 from many a wealthy congregation. (Applause.) Another highly satisfactory feature of the report was that while the whole work was so immensely increased, while the amounts received had risen from £27,700 to £46,035, and while the distributing of these large sums entailed so very great an amount of office work, investigation, and calculation, the whole expenses for machinery was kept so low. This year it was £1,675 as against an average for the last twenty-three years of £1,585, and it only amounted to 3·638 per cent. of the receipts. (Applause.) Another very satisfactory feature was seen in the fact that the operation of the Hospital Sunday Fund had tended very decidedly to the improvement of the methods of many of the hospitals. He was himself very much averse to using a charity for the purpose of prying and interfering. This great charity was not so used, but it would be seen on reference to a paragraph on page 8 of the report that the Committee of the Distribution required very careful keeping of accounts and balance-sheets on the part of the hospitals and dispensaries as a preliminary to a grant being made. (Applause.) In conclusion he referred appreciatively to the help so freely given to the Fund by the public press.

Mr. F. H. Norman, in seconding, suggested that the increase in the number of contributing congregations and the amounts given by them should, in face of the largely-increasing population, have been greater than four (*i.e.*, from 1,807 n 1895 to 1,811 in 1896) and £2,130 (*i.e.*, from £38,371 to £40,502) respectively.

The resolution was carried unanimously.

On the motion of the Chairman, seconded by the Rev. C. H. Grundy, the laws of the constitution which had been in force during the past year were readopted.

Mr. Henry C. Burdett proposed "That the Councilfor the year 1896, together with the retiring members, be re-elected for the year 1897, with the Right Rev. Bishop Barry, D.D., rector of St. James's, Piccadilly, in place of the Rev, the Hon. E. Carr Glyn, M.A., appointed Lord Bishop of Peterborough; H. Cosmo O. Bonsor, Esq., M.P., in place of Mr. Alderman G. Faudel-Phillips, now Lord Mayor; the Hon. Sydney Holland in place of Admiral the Hon. F. Egerton, deceased; and that it be deputed to the Council to fill in any vacancy that may arise during the year." It had been said, not in recent years, but formerly, that there was one objection to Hospital Sunday Fund, and that objection was that its Council was not representative of hospital opinion. He ventured to think that the mere wording of the resolution, and the names of those members who were going off, as well as those who were coming on, the Council, formed the best answer to a criticism of that kind. The Hospital Sunday Fund was able to attract to its Council the most active men in the hospital world at the present time, and each year as vacancies occurred the Executive Committee took the utmost pains to see that the Council secured the services of the most active and vigorous men interested in our medical institutions. In conclusion, he expressed, on behalf both of himself and of his colleagues on the Council, the deep regret they felt at the retirement from that body of Lord Knutsford, who had recently intimated his intention of resigning on account of old age. (Applause.)

The Chief Rabbi seconded, and reminded his hearers of the fact that next year was, in addition to being the diamond jubilee of the Queen's reign, the silver jubilee of the Fund, which would then have been established 25 years. He hoped, therefore, that next year they would be able to order their committee to distribute a larger amount than ever—an amount more nearly approaching their ideal sum of £100,000. (Applause.)

On the motion of the Rev. Canon Graham, seconded by the Rev. Canon Fleming, June 20th was fixed for the date of Hospital Sunday in 1897.

A vote of thanks to the chairman terminated the proceedings.

### POOR LAW OFFICERS' SUPERANNUATION.

A well-attended general meeting of the Hospitals Association was held at the Society of Arts, John Street, Adelphi, W.C., on Monday, 14th inst., Mr. Edmund Boulnois, M.P., in the chair.

The Chairman having briefly opened the meeting,

Mr. H. Elwin Harris, Medical Superintendent of St. Saviour's Infirmary, read an excellent paper on "The Poor Law Officers' Superannuation Act." He dealt with the subject chiefly as it affected nurses under the Poor Law, and prefaced his remarks with the statement that, except so far as it concerned subordinate officials, he was as much in favour of the Act as any other Poor Law officer. After detailing the main provisions of the Act, Mr. Harris said that, although on paper it seemed most just, it pressed very unfairly on nurses. The two chief reasons for this were—first, that nurses did not remain for long in the service, and contributions were not returnable; and, secondly, that the age of retirement, both compulsory (65) and voluntary (60), was too great. It was a moral and physical impossibility for nurses in infirmaries or workhouses to reap any benefit from the provisions of the Act. Their work was both trying and laborious, most of the cases were chronic and devoid of professional interest, and several were helpless and required much lifting. A woman approaching the age of sixty, though in good bodily health, would be physically incapable of doing this. The Local Government Board would not increase the staff because of the advancing age and infirmity of its members, and the patients would consequently suffer. It was the absence of permanent bodily infirmity with the presence of inefficiency which would, if the Act remained in its present form, be a most difficult matter to cope with, and would of necessity lead to the deterioration of workhouse nursing. Then, too, all the Metropolitan infirmaries were training schools for nurses, and, therefore, it was essential that the majority of the head nurses, from whom the probationers must necessarily learn the details of nursing, should be comparatively fresh from hospital, up-to-date, and in possession of the latest views on matters connected with nursing. In support of the justice of the view that it should be optional for nurses and subordinate officials to contract out, Mr. Harris said that out of 3,320 officers engaged under the Metropolitan Asylums Board who came under the provisions of the Act, no less than 2,340, or 70 per cent., had availed themselves of the opportunity of contracting out. It could not be assumed that 70 per cent. of the Asylums Board officials were so dense that they did not know when a good thing was offered them. The Act could not be the uniform boon that its promoters would have people believe, and if it remained in its present form the immediate successors of these 2,340 officials would have to submit to the percentage deduction of their salaries whether they liked it or not. It might be said that they would enter the service with their eyes open, but this was no argument at all. These people must earn their living, and he asked whether it was right and just to take advantage of their necessity, and compel them to contribute to a fund out of which their chance of deriving any benefit was most remote.

### The Discussion.

The discussion was commenced by

Dr. Rowland Humphreys, who said it was a great injustice that nurses, under an Act which was supposed to be for their benefit, should be compelled to supply a sum of money which they would never get back again, and expressed his indignation that such an Act should have been allowed to pass in spite of the protests of those who had the interests of nurses at heart.

Mr. Jackson Hunt, Chairman of the St. Marylebone Board of Guardians, felt most strongly that all who subscribed to a superannuation fund should have a reasonable prospect of benefiting by that fund. In his opinion, nurses would not benefit under the Act, for by far the greater number of nurses who entered Poor Law infirmaries did not intend to make Poor Law nursing the profession of their lives. As bearing out this contention, he said that out of 197 nurses who had left the St. Marylebone Infirmary since 1884, only 20 were now in the service of the Poor Law or Metropolitan Asylums Board, and would therefore be in a position, if they stayed long enough, to come upon the Superannuation Fund for a pension. He supplemented the statistics as to "contracting out" in the Metropolitan Asylums Board's service, given by the reader of the paper, with the following: Out of 877 nurses in the service of that Board 755, or 84 per cent., had "contracted out." It was a curious fact that at the St. Marylebone Infirmary, out of 72 nurses (including matrons) 59, or 82 per cent., had "contracted out." Very few nurses remained in the service for even ten years. Out of 364 Metropolitan Asylums Board nurses in 1894 158, or 44 per cent., left with less than two years' service, a fact from which it was fair to deduce that a very much larger number would leave with less than ten years' service.

Dr. F. S. Toogood, Medical Superintendent, Lewisham Infirmary, thought that under Section 8 of the Act all probationers trained at present at Poor Law infirmaries would be entitled to the return of the whole of the contributions made by them under that Act. As showing the way in which nurses had received the Act he said that at his infirmary only one nurse had availed herself of its provisions, and she had sustained grievous bodily injury in the discharge of her duty.

Dr. Goodall did not see how those people who were going to enter the Poor Law service in future had cause to complain of the provisions of the Act. How statistics, showing the number of nurses "contracting out," affected the general question as to whether those who intended to join in the future were hurt by the Act or otherwise he also did not see.

Mr. J. H. Rutherglen, Clerk to the St. Mary Abbotts Infirmary, defended the Act and its promoters at some length. The matter dealt with in the Act was not new. It had been discussed, it had been the subject of memorials to the Local Government Board—as far back as 1886 a memorial was signed by 8,000 Poor Law officials of all degrees in favour of a superannuation scheme—and when the bill was before Parliament last session dozens of petitions in favour of the Bill had come through his hands signed by nurses, kitchen-maids and female servants in the service throughout the country. He held that the Act was just as applicable to every female officer in the service as it was to male officials, and produced figures in support of this. Out of 18,400 officers who were recognised as coming on superannuation, 4,600, or as nearly as possible 25 per cent., were nurses, matrons, school mistresses, and other female officers. Besides this, out of 100 officers who were superannuated in 1895 under the Act, 61 were males and 39 females. Could it, he asked, be maintained in the face of figures such as those that the female officers were not as likely to come on for superannuation as the male? Having explained how it happened that, when the Act was passed, the amendment proposed by Mr. Boulnois allowing all nurses present and future to contract out if they wished, had not been inserted, Mr.

Rutherglen read the principal clauses of a Bill which it was proposed to introduce next session carrying out this amendment.

Mr. J. K. Musto, Master of the St. Mary, Islington, Workhouse, said the Committee which had charge of the Superannuation Bill was a most one-sided one, composed of officials of one class. He hoped a thoroughly representative committee would be appointed to carry out the Bill Mr. Rutherglen had read.

Miss Gibson, Matron of the Birmingham Royal Infirmary, said that the chief point—the interest of the patient—had been lost sight of entirely. It was impossible that work-house infirmaries should be nursed by elderly women. The nurses must be young women, women who were enthusiastic about and fond of their profession. There was no question that younger nurses were the most efficient, and had a better effect both morally and physically on the patients than nurses who had become wearied with years of monotonous work and were tired of nursing, but who were going on with it because they would in time get a pension. The only conclusion, therefore, was that a nurse should not remain permanently in the service. Another point which was not apparently realised by those in favour of the Act was the fact that nurses in the service were a special class—they were nurses first, and their being in the service of the Poor Law was merely an accident of their position.

Miss Wilson, hon. secretary of the Workhouse Infirmary Nursing Association, said that if Mr. Rutherglen, as the principal promoter of the Bill, had been able to meet the views expressed to him before the Act was passed a great deal of trouble would have been saved. The amending Bill which he had read included, as a matter of fact, more than had been asked for previously. After a eulogistic reference to the Royal National Pension Fund for Nurses as a means of provision against old age, Miss Wilson said that if Mr. Rutherglen would move a resolution regarding the amending Bill she would have the greatest possible pleasure in seconding it.

Mr. Rutherglen accordingly moved: "That this meeting approves of the Bill proposed to be introduced into the ensuing session of Parliament to enable nurses appointed in future to contract out of the provisions of the Poor Law Superannuation Act, 1896, and pledges itself to render every assistance to secure its passing into law."

Miss Wilson seconded, saying that she thought it would be a very great advantage if a committee, representative of the interests of nurses, were formed to help forward the Bill.

Mr. Preston, Clerk to the Hampstead Guardians; Mr. Parkinson, Master of St. Olave's Union; and Dr. D. M. Forbes, Medical Superintendent of St. Leonard, Shoreditch, Infirmary, carried on the discussion. The latter said that in twenty-five years at the Shoreditch Infirmary only four nurses had received pensions.

The Chairman closed the discussion. After thanking Mr. Harris for his paper, he said that although they had a remedy for the existing state of affairs in the Bill Mr. Rutherglen had read, they must not be content with that. They must urge the Boards of Guardians to insist that the Local Government Board should take steps in the direction indicated in that Bill. If the Local Government Board found that there was a universal and overwhelming feeling amongst nurses against being included under the Poor Law Superannuation Act, 1896, they would not only help forward the Bill, but probably would take steps to help the nurses themselves.

The resolution was carried unanimously.

Mr. Henry C. Burdett proposed a vote of thanks to the Chairman for presiding, and to Mr. Harris for his excellent paper. It was most important, he said, as bearing upon the efficiency of the Poor Law service, that all should recognise from what had occurred that evening that there was now a union of all branches of the service. One result of the meeting, he hoped, would be the removal of misconceptions, and the getting through Parliament of the best Bill possible in the interests of all classes.

Mr. J. H. Rutherglen seconded, and the resolution was carried.

A very brief reply by the Chairman concluded the meeting.

### SURGICAL AID SOCIETY.

The annual meeting of the Surgical Aid Society was held at the Cannon Street Hotel on Wednesday, 9th inst., the Lord Mayor in the chair. Amongst those present were: Sheriffs Ritchie and Rogers, Sir Francis H. Jeune, the Mayor of Croydon, and Sir Joseph Ewart, M.D.—The Lord Mayor, in moving the adoption of the report and accounts, spoke very highly of the work of the society, which, he said, was very well thought of at the Mansion House. A point in its administration which commended it to him was the fact that it only had about £10,000 invested, although it spent over that sum each year in relief. In his opinion, the present generation should relieve present suffering to the fullest extent that they could, and should use every effort to secure the largest amount possible being placed at the disposal of charity. They should not hoard their money, for those who came after them would, he was sure, recognise their obligations as fully as the present generation did.—The resolution was seconded by Mr. Sheriff Rogers and carried. Votes of thanks to the treasurer, he committee, and the surgeons were passed, and the officers te-elected. A vote of thanks to the Lord Mayor for presiding rconcluded the proceedings.

